# Exploring the Relationship Between Chinese EFL Students' Grit, Well-Being, and Classroom Enjoyment

**DOI:** 10.3389/fpsyg.2021.762945

**Published:** 2021-10-29

**Authors:** Peng Yang

**Affiliations:** College of Foreign Languages, Pingdingshan University, Pingdingshan, China

**Keywords:** classroom enjoyment, EFL students, students' grit, students' well-being, EFL

## Abstract

It has been documented that grit plays an indispensable role in the process of language learning and teaching. It is postulated that gritty people are more able to become involved in classroom practice and remain motivated even in light of challenges; however, what remains vague is the interplay of grit, well-being, and classroom enjoyment. To this end, 335 male and female Chinese EFL (English as a Foreign Language) learners who were studying English in 28 universities took part in this study. They completed three questionnaires including the grit scale questionnaire (Grit-S), foreign language enjoyment scale, and PERMA well-being scale. The Pearson coefficient of correlation was run to investigate the first research question of the study while, after checking the preliminary assumptions, for the second research question a multiple regression analysis was used. The findings of the study demonstrated that there is a positive relationship between learners' grit and enjoyment, and high degrees of enjoyment were interrelated to high degrees of grit. The findings of the study also signified that grit significantly predicted students' well-being and was also a predictor of classroom enjoyment. Finally, some implications and recommendations have been offered for language teaching stakeholders in educational settings.

## Introduction

The instructive research domain was preoccupied with inspecting negative emotions such as boredom, apprehension, stress, and foreign language classroom anxiety in classes (Horwitz et al., 1986; MacIntyre and Gardner, 1989; Shaw et al., 1996; Harris, 2000; Horwitz, 2000, 2001; Rodríguez and Abreu, 2003; Tran, 2012; Capone et al., 2019; Fathi and Derakhshan, 2019; Chen et al., 2021; Derakhshan et al., 2021). But, considering the negative aspects is only half of the issue, so a new approach, positive psychology (PP) in educational settings, has grown throughout previous decades and is welcomed by scholars (Mercer et al., 2018; Chen and Padilla, 2019; De Ruiter et al., 2019; MacIntyre et al., 2019; Wang et al., 2021), and the eminence of affectivity in both second and foreign language instruction research has been at the center of attention of many researchers lately (Fathi and Derakhshan, 2019; Derakhshan, 2021; Pishghadam et al., 2021; Xie and Derakhshan, 2021; Yazdanmehr et al., 2021; Zeng, 2021). PP hunts for congruence between conventional psychology, with its emphasis on reviewing problematic manners such as anxiety and stress, but moves the route of the study to human powers and constructing psychological capitals that embrace the procedures and circumstances that result in pleasure, enjoyment, and efficacy (Seligman, 2011). That is to say, PP directs attention on specifying human assets and how they can construct psychological sources to succeed and flourish in their daily life (Seligman and Csikszentmihalyi, 2000). Through positive emotions, students can process everything in their classroom situation better and they come to be more conscious of language input, leading to their success (Fang and Tang, 2021).

A dominant concept scrutinized in the PP realm is that well-being that is a multifaceted theory including positive emotions, enjoyment, engagement, positive interactions, meaning, and success or achievement (Seligman, 2011). One motive for the improvement of well-being is that it considers more than just feeling well. People who are pleased with their lives are more efficacious and more involved (Peterson et al., 2005). Those who described high levels of well-being encounter positive emotions more regularly through learning tasks, which are considered as an assessment and appraisal of the whole learning procedure (Jing and Yu, 2015; Yuting et al., 2020).

One of the positive emotions which has received increasing attention in language education is a specific kind of enjoyment that is theorized as the state of experiencing enjoyment in learning L2 (Li C. et al., 2018; Jiang and Dewaele, 2019). Enjoyment is a construct that echoes the evolving field of PP in general and its underlying theories such as the Broaden-and-Build Theory (Fredrickson, 2001) and the Control-value Theory (Pekrun and Linnenbink-Garcia, 2012) in particular. Based on the former theory, enjoyment boosts the structure of bases in language education with its positive influence on developing a person's perception (MacIntyre and Gregersen, 2012), and in line with the latter theory, enjoyment is a constructive, stimulating activity-focused emotion that affects students' educational success (Piniel and Albert, 2018). Enjoyment deals with other encouraging emotions to expand one's temporary range of opinions and actions that shape their emotional resiliency, among other personal factors (Oxford, 2015). Enjoyment is defined as a good emotional state broadening away from oneself to achieve something novel or even surprising; particularly in dealing with challenging tasks that enhance personal growth and enduring well-being (Csikszentmihalyi, 2014). Enjoyment enhances language education since it motivates students to act in class and to look for strange phonological and cultural domains (Dewaele and MacIntyre, 2014), so it is meaningfully correlated with language accomplishment (Dewaele and Dewaele, 2017; Dewaele and Alfawzan, 2018; Saito et al., 2018).

Another positive inner construct is grit; a constructive spiritual construct, defined as perseverance and passion for striving toward longitudinal objectives (Duckworth and Quinn, 2009), grit has been experimentally evaluated as a prospective preliminary of learners' success through a selection of academic fields (Rojas et al., 2012; Bowman et al., 2015; Sturman and Zappala-Piemme, 2017; Li J. et al., 2018). Grit is associated with motivation, as Ryan and Deci (2000) proposed; those who have intrinsic motivation in the process of their works and have certain, specific goals are more interested, confident, and find more enjoyment, which sequentially displays in higher presentation, perseverance, resourcefulness, and overall well-being (Ryan et al., 1995). Therefore, grit is a type of disposition that mirrors the quantity of energy that persons use in the development of endeavoring objectives that contributes to their individual growth (Duckworth, 2016) and it is one of the significant predictors to a learner's educational success, that is, learners who work hard are interested in what they do, and are expected to be more able to deal with the obstacles of language learning, which leads to better performance (Dweck et al., 2014). Indeed, in PP, grit is deemed to have an even bigger role (MacIntyre et al., 2019) and is supposed to be a basic element in language education (Keegan, 2017). In the collected works, it is known as a beneficial quality for those who tend to flourish in learning as it has been interwoven with greater levels of engagement and theoretical efficiency (Cross, 2014; Credé et al., 2017; Muenks et al., 2017; Reed and Jeremiah, 2017; Hodge et al., 2018). In this vein, gritty learners are those that take advantage of a great amount of energy in endurance related to their goals and are likely to concentrate on accomplishing objectives that exemplify their interests (Duckworth and Gross, 2014; Schmidt et al., 2017).

Moreover, grit is a non-cognitive and constant higher-level disposition including two frameworks, namely consistency of interest and perseverance of effort and these frameworks of grit can result in a variety of consequences for those with parallel cognitive skills (Duckworth et al., 2007). There is a comprehensive review of literature that investigates the role of grit in language education, though it is in a quite early stage (Keegan, 2017; Wei et al., 2019, 2020). It is only recently that scholars have started to inspect this construct in general, and in language settings in particular, having the agenda to comprehend the role of grit with respect to well-being and enjoyment, in spite of the noticeable function in language education of grit in contrast to other issues (MacIntyre et al., 2019). The role of grit and its predictive impacts on educational accomplishment has been evidenced by several experts (e.g., Duckworth, 2017; Datu et al., 2018; Musso et al., 2019; Alhadabi and Karpinski, 2020; Meyer et al., 2020); however, grit has been interrelated to numerous indicators of well-being in multicultural research, pleasure, and life gratification (Kleiman et al., 2013; Jin and Kim, 2017). Studies have established that grit is significantly related to desire (Sigmundsson et al., 2020), resilience (Calo et al., 2019; Shakir et al., 2020), well-being (Moen and Olsen, 2020), and enthusiasm (Steinmayr et al., 2018; Karlen et al., 2019).

Although the abovementioned studies have examined grit and its effect on various variables, first, there are not sufficient studies that have scrutinized grit's relationship with classroom enjoyment and well-being among EFL (English as a Foreign Language) learners and secondly, there are quite a few studies that have approved predictors of grit. As a result, there was a gap that needed to be inspected which could aid literature in the generalizability of grit in the language education domains. Furthermore, this study aims to increase understanding concerning the predictive properties of grit upon classroom enjoyment and well-being.

Inspired by the lacuna in the literature relating to the predictor role of grit and the educational prominence of the variables discussed in the introduction section, the subsequent research questions were articulated in the present study.

Q_1_. Is there any significant relationship between Chinese EFL student's grit, well-being, and their classroom enjoyment?
Q_2_. Does grit predict Chinese EFL students' well-being and classroom enjoyment?

## Literature Review

### Well-Being

Emotions have a critical contribution in the EFL context (MacIntyre et al., 2019). PP scholars have conceived many principles to enlighten the paradigms of well-being (Huppert and So, 2013; Rusk and Waters, 2015; Butler and Kern, 2016). Two approaches exist in well-being (Cooke et al., 2016), first, the hedonistic approach that centers on desire and pleasure, in which the most crucial factor is well-being, encompassing life gratification and the presence of positive feelings (D'Mello and Graesser, 2012; MacIntyre and Mercer, 2014). The second is the eudaimonic approach that concentrates on more parts of life, even though they differ from the important basics that regulate well-being, while PP integrates the hedonic and eudaimonic together.

In addition, the conception of well-being is defined by Seligman (2011) with his five important aspects all coming into the positive continuum of psychological well-being named positive emotion, engagement, relations, meaning, and achievement as PERMA. Positive emotions including pleasure, optimism, and well-being all fall into the hedonic continuum of emotional states that function as signs of prosperity, though they can help a person to flourish and all and can be taught and improved (Fredrickson, 2001). Engagement is typically labeled as a form of flow, or deep involvement, which aims to motivate inherently during task completion (Dixson et al., 2017; Derakhshan, 2021). Goal setting, observation, and success contribute to well-being throughout the life sequence (Heckhausen et al., 2010). Positive relationships allude to the sense of being socially incorporated, acknowledged, and reinforced by others and being pleased with an individual's social link. Social support has been connected to both positive spiritual and physical well-being consequences and well-being generally (Cohen, 2004; Karademas, 2006; Greenier et al., 2021). Meaning refers to the idea that an individual's life has perseverance and a route in the lifecycle, and to feel attached to something greater than oneself. Meaning has been associated with well-being consequences and also with positive emotions in diverse age groups (Cotton Bronk et al., 2009). Accomplishment is usually linked to goal setting, growth, and having an ability to achieve, thus striving for well-being (Croom, 2015).

Indeed, taken from the principle of PP, and within constructive education, Seligman's PERMA model (Seligman, 2011) was assimilated into the curriculum to improve the well-being of learners and assist them to flourish and improve their achievements. PERMA is a model that is consistent and authorized by many studies and it is also multidimensional that provides more information on the well-being of respondents.

### Enjoyment

Enjoyment can be defined as an instance of progressive accomplishment emotions (Pekrun, 2006). Students who come across enjoyment recognize the mechanism of tasks they are engaged in and feel those tasks results as remarkable (Pekrun et al., 2007). In this way, classroom enjoyment can be supposed as the preference practiced when students admire learning the subject matter (constructive review) and feel proficient in managing and finalizing the tasks they are confronted with (control). Accordingly, enjoyment is thought to be of the greatest prominence for the following feeling of fulfillment, which matches educational accomplishment (Ainley and Hidi, 2013; Piechurska-Kuciel, 2017; Dewaele and Li, 2021). Considering enjoyment as a multidimensional paradigm, it holds five mechanisms including the emotional, intellectual, motivational, communicative, and physical (Hagenauer and Hascher, 2014; Elahi Shirvan and Taherian, 2020), in which attention has been drawn mainly to the first three. Addressing foreign language settings, it is rational to consider that the emotional aspects of enjoyment refer to the feeling of enjoyment practiced during learning a language, while the intellectual one refers to a constructive appraisal of the circumstances in which the language learners are involved in. Besides, classroom enjoyment might be designated as the sense of enthusiasm or an impromptu enjoyment taken from involvement in stimulating language tasks (emotional component), which provokes students' interest and engenders attentiveness (intellectual component). Consequently, it is practical to consider that enjoyment arouses enthusiasm in students in the language classes, as it may have a key role in the intellectual procedures which are influential for learning—on the one hand, language learning, on the other hand, e.g., intensified thoughtfulness, retention, and problem solving (Fredrickson, 2004; Linnenbrink-Garcia and Pekrun, 2011; Oades-Sese et al., 2014).

### Grit

Grit, as a non-cognitive distinct trait that impacts language education consequences, has a fundamental position in individual accomplishment (Duckworth et al., 2007; Duckworth and Quinn, 2009), and emotional paradigms like sadness and enthusiasm (Steinmayr et al., 2018; Datu et al., 2019). Grit is described as the aptitude to endure difficulty while preserving desire for long-term objectives (Cross, 2014; Eskreis-Winkler et al., 2014; Wolters and Hussain, 2015; Howard et al., 2019). Duckworth et al. (2007) pinpointed that grit is the capability to draw on concerns and overcome troubles while also enduring grits to struggle with setbacks. The grit contains trait-level mechanisms of perseverance and passion for long-standing purposes (Duckworth and Quinn, 2009; Duckworth, 2016). The perseverance of effort refers to the intensive desire and grit to accomplish a goal, and related grit with resiliency, determination, resolution, and carefulness, and it defines the extent to which individuals can withstand obstructions and challenges, (Duckworth et al., 2007; Datu et al., 2019). The latter is the passion for long-standing goals, referring to the capability of an individual for sustaining attention for a prolonged period, and also refers to the extent to which persons regularly place emphasis on accomplishing their long-term goals (Duckworth et al., 2007; Datu et al., 2019).

Gritty people display benefits of learning attainment, the permanency of engagement, and perseverance through challenging teaching (Eskreis-Winkler et al., 2014). On the part of scholars, there are plenty of signals verifying the momentous relationship between grit and positive results (Duckworth and Quinn, 2009; Eskreis-Winkler et al., 2014). For instance, persons who have more grit receive meaningfully higher marks, both in school and at college, and continue to accomplish more innovative stages of learning than those who show lower degrees of grit, while greater degrees of grit determine better accomplishment in extra-curricular tasks (Duckworth et al., 2007; Duckworth and Quinn, 2009) and have considerably higher degrees of school achievement (Eskreis-Winkler et al., 2014).

Furthermore, grit tries to seize the concepts of resilience, carefulness, self-control, and persistence to one extent, perceptions that have previously been maintained to be primary to educational accomplishment (Bashant, 2014; Teimouri et al., 2020). Within an educational setting, resilience has been determined as the progression of, the capability for, or consequence of positive adjustment in spite of stimulating or frightening situations, which is deemed as the main element encouraging accomplishment in learners (Martin, 2002). The disposition facet of conscientiousness is usually supposed to be a moderately permanent feature; nevertheless, there is continuing debate about this issue and conscientiousness comprises the notions of trustworthiness and caution and has a robust encouraging connection with academic results (Mischel, 2013). Self-control seizures the dimensions to control thoughtfulness, feeling, and manners in the occurrence of attraction (Duckworth and Gross, 2014).

Besides, as stated by Sariçam et al. (2015), grit is a central and indispensable issue in accomplishing social-expressive growth in all other aspects of life and can be deemed as a social-expressive ability of ethical worth. Inquiries have informed that grit is interrelated to success, self-efficacy, self-regulation, metacognition, despair, and anxiety (Bowman et al., 2015; Wolters and Hussain, 2015; Ozhan and Boyaci, 2018; Lee, 2020; Fathi et al., 2021). Grit involves involvement in a superordinate aim that stands on the highest of a pyramid while lower-level objectives are firmly associated and intend to move in the direction of the superordinate aim (Duckworth and Gross, 2014).

Vainio and Daukantaite (2016) have done a study on adults in which they examined the predictive role of grit in estimating life satisfaction and well-being. They concluded that individuals who think of long-term objectives were much happier across the course of their life, more self-confident, and felt more optimistic about their interactions, perseverance, and improvement. Likewise, Lee (2020) tried to study the implication of grit and the purpose of commitment in the association between grit and educational success, so the grit, engagement, and educational efficiency of 395 Australian learners was measured. The result of the study revealed that there is no difference in grit concerning genders and that there was a relationship between grit, engagement, and educational attainment. Moreover, engagement facilitated the correlation between grit and success, indicating that a person with greater grit is more ready to have greater engagement that causes better academic efficiency. Besides, Wei et al. (2019) aimed to scrutinize the influence of grit on language presentation among 832 learners. An intermediated self-control model was built to measure the influential function of enjoyment and the regulating function of the classroom setting in the connection between grit and language performance. The results of the study through correlation and regression analyses specified that grit positively affected language performance. Besides, language enjoyment interposed the correspondence between grit and language performance, and classroom setting moderated the correlation concerning grit and enjoyment, and regarding grit and language presentation.

## Method

### Participants

Using the convenience sampling method, 335 respondents from 28 universities that are located in China, were invited to take part in the current study. All the participants were university EFL students from five provinces of China (Henan, Hubei, Anhui, Shandong, and Shaanxi). The respondents were undergraduate and post-graduate students aged between 18 and 25 years old, and they were heterogeneous in terms of gender, with 87 men (25.97%) and 248 women (74.03%) students. All the respondents in this survey were reassured that their information would be kept in confidence and be utilized for research purposes only.

### Instruments

#### Grit Scale Questionnaire (Grit-S)

The researcher used the Grit-S as a relatively short Likert scale eight-item questionnaire including two scales, namely consistency of interest and perseverance of effort. This scale was developed and validated by Duckworth and Quinn (2009) and is called the Grit Scale questionnaire, where items are rated on a 5-point scale from 1 = not at all like me, to 5 = very much like me, to measure how gritty a person is. Prior research (Duckworth and Quinn, 2009) reported satisfactory to good reliability for the Grit-S, with Cronbach α values with a range of 0.73 to 0.83. Cronbach's α value for the stabilities of interest subscale was somewhat better than the ones for the perseverance of effort subscale (ranging from 0.73 to 0.79 and 0.60 to 0.78 correspondingly).

#### Foreign Language Enjoyment Scale

To identify the learners' level of enjoyment, the researcher used the foreign language enjoyment scale containing 21 items developed by Dewaele and MacIntyre (2014). These items mirror both the societal and private aspects of language enjoyment (Dewaele and MacIntyre, 2014). Respondents were asked how much they agreed or disagreed with the sentences on a 5-point Likert scale ranging from “strongly disagree,” to “strongly agree.” The internal consistency of this scale was about 0.88 measured through Cronbach alpha (Mierzwa, 2019). It is worth mentioning that the reliability of this scale calculated through Cronbach alpha is 0.83.

#### PERMA Well-Being Scale

The researcher used PERMA scale established by Butler and Kern (2016) to measure the conception of Martin Seligman's five aspects of well-being which contains 15 items, three items in each dimension. The PERMA-Profiler subscales have confirmed satisfactory reliability and validity (Butler and Kern, 2016).

### Data Collection Procedures

For the purpose of this study, 335 male and female Chinese EFL students with an age range of 18–25 took part in the online survey via we-chat. The package of three questionnaires on grit-s, foreign language enjoyment, and PERMA well-being was given to them online, due to problems presented by the Covid-19 pandemic, with the intent that the students were asked to ask questions in the case of any problems. It is worth mentioning that they were provided with sufficient knowledge and information on the process of filling out the questionnaires to provide valid data. They were assured that the data gathered by the questionnaires was only for the research objectives. In order to motivate the students to answer the questionnaires, the researcher provided them with the opportunity to get feedback on the outcomes of each scale through e-mail. At the final step, the statistical procedures were conducted to inspect the research hypotheses.

### Data Analysis

To observe the probable relationship between the variables of the study, the Pearson product-moment correlation coefficient was used. According to Plonsky and Oswald (2017), running multiple regressions as an alternate to *t*-test and ANOVAs is better to represent individual differences characteristic of data when the study is multivariate. Thus, multiple regression analyses were employed to answer the second research question to check the predictor role of grit on these two variables, namely enjoyment, and well-being.

## Results

### Preliminary Analyses

The data were investigated through Pearson's Product-moment correlation coefficient and linear regression which both had several assumptions such as, normality, linearity, and homoscedasticity. First, by inspecting the boxplots for each variable, twelve cases (Case No 52, 82, 180, 183, 192, 200, 224, 230, 255, 302, 315, 324) which showed characteristics of outliers were excluded. Then the normality was probed using skewness/kurtosis values. Table 1 reports the descriptive statistics of the results.

**Table 1:** Descriptive statistics.

	***N***	**Min**.	**Max**.	**Mean**	**SD**	**Skewness**	**Kurtosis**
Grit	335	8	39	29.57	5.296	−1.633	3.707
PERMA	335	25	68	53.03	8.800	−0.938	0.905
Enjoyment	335	45	95	78.27	7.966	−1.271	2.627

**Figure 1:**
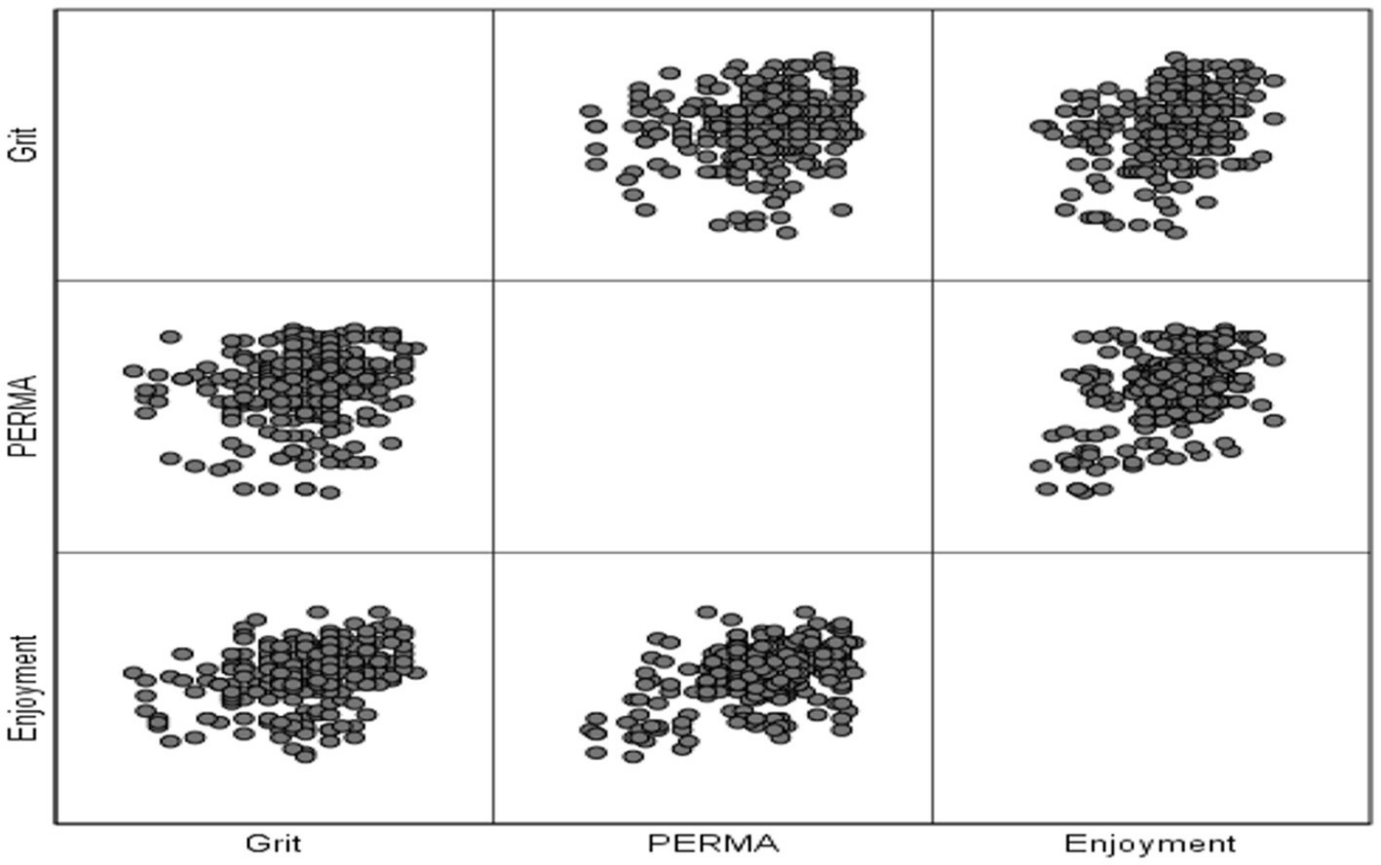
Multiple scatterplot of the relationships between variables.

As demonstrated in Table 1, the skewness and kurtosis values fell within the variety of ±1.96, indicating normalcy of distribution for all data sets (Tabachnick and Fidell, 2013). The next assumption to be checked was the linearity of the relationship between pairs of variables and homoscedasticity. To check these assumptions, a multiple scatterplot was shaped (Figure 1).

Through examining Figure 1, it can be concluded that the interactions among these variables are not basically non-linear. As it is apparent, there is not a U-shaped or curvilinear form of distribution. Consequently, the linearity of associations can be established. Moreover, the scattering of scores was not funnel-shaped, i.e., wide at one end and narrow at the other; consequently, the assumption of homoscedasticity was assured for these items.

### Answering the Research Questions

Having all the assumptions in place, running a Pearson correlation to retort the first research question was legitimized. Table 2 displays the upshots of the Pearson correlation tests.

**Table 2:** Pearson correlations among the variables.

		**Grit**	**PERMA**	**Enjoyment**
Grit	Pearson correlation	1		
	Sig. (2-tailed)			
	*N*	323		
PERMA	Pearson correlation	0.188**	1	
	Sig. (2-tailed)	0.001		
	*N*	323	323	
Enjoyment	Pearson correlation	0.337**	0.455**	1
	Sig. (2-tailed)	0.000	0.000	
	*N*	323	323	323

** *Correlation is significant at the 0.01 level (2-tailed)*.

Consistent with the outcomes of the analysis displayed in Tables 3, 4, it was proved that there was a positive relationship between grit and PERMA, *r* = 0.188, *n* = 23, *p* < 0.01, and high degrees of PERMA were related to high degrees of grit. As stated by Cohen (1988), this indicated a small effect size (95% confidence intervals: 0.081 to 0.291). Moreover, the results displayed a positive relationship between grit and enjoyment, *r* = 0.337, *n* = 323, *p* < 0.01, and high degrees of enjoyment were linked to high degrees of Grit. As stated by Cohen (1988), this indicated a small effect size (95% confidence intervals: 0.237 to 0.43).

**Table 3:** Summary of the regression models.

**Model**	**R**	**R Square**	**Adjusted R Square**	**Std. Error of the Estimate**	**Durbin-Watson**
1^b^	0.188^a^	0.035	0.032	8.461	1.083
2^b^	0.337^a^	0.114	0.111	6.464	1.685

*Predictors: (Constant), Grit. a. Dependent Variable: PERMA. b. Dependent Variable: Enjoyment*.

**Table 4:** Regression results: ANOVA.

**Model**		**Sum of squares**	**df**	**Mean square**	***F***	**Sig**.
1	Regression	844.491	1	844.491	11.798	0.001
	Residual	22977.200	321	71.580		
	Total	23821.690	322			
2	Regression	1718.979	1	1718.979	41.136	0.000
	Residual	13413.783	321	41.787		
	Total	15132.762	322			

The next question required running linear regressions. Two linear regressions were run separately to test the predictability of EFL students' well-being and classroom enjoyment by grit. Table 3 summarizes the two regression models.

As described in Table 3, for the first model (well-being as predicted variable), R came out to be 0.188 and R^2^ came out to be 0.035. This implies that the model describes 3.5% of the variance in the total score of well-being. The difference between the observed and adjusted R^2^ (0.035 – 0.032 = 0.003) indicated that the observed predictive power had 0.003 (0.3%) differences with the population index, which indicates high generalizability. Moreover, the Durbin-Watson (DW) index of 2.243 showed that the assumption of independence errors was assured. As stated by Tabachnick and Fidell (2013), DW files between 1 and 3 are satisfactory.

In the second model R and R^2^ came out to be 0.337 and 0.114, respectively. This shows that the model can explain 11.4 percent of the variance of the total writing scores in the descriptive writing test. The variance between the observed and adjusted R^2^ (0.114 – 0.111 = 0.003) indicated that the observed predictive power had 0.003 (0.3%) differences with the population index, which indicates high generalizability. The DW index of 1.618 specified that the supposition of independence error was also met for this model. Table 4 displays the effects of ANOVA as an output of regression.

Table 4 inspects the statistical significance of the regression models. The findings for the first model (*F*_(1,321)_ = 11.798, *P* < 0.05) indicated that grit significantly predicted students' well-being. It also was the significant predictor of classroom enjoyment, as indicated by the results for the second model (*F*_(1,321)_ = 41.136, *P* < 0.05). Finally, the regression coefficient for the two models is reported in Table 5.

**Table 5:** Regression results: coefficients.

**Model**		**Unstandardized coefficients**		**Standardized coefficients**	***t***	**Sig**.
		**B**	**Std. error**	**Beta**		
1	(Constant)	41.507	3.507		11.837	0.000
	Grit	0.395	0.115	0.188	3.435	0.001
2	(Constant)	61.910	2.679		23.107	0.000
	Grit	0.564	0.088	0.337	6.414	0.000

Table 5 shows the regression coefficients which can be implemented to articulate the regression equations, as presented below:


$$\begin{array}{l} \text{ Well}-\text{being}=\left(\text{Grit}{\;}^{*}\;0.395\right)\;+\;41.507\\ \text{Classroom enjoyment}=\left(\text{Grit}{\;}^{*}\;0.564\right)\;+\;61.91 \end{array}$$

In the first model, the beta value of 0.188 indicated that one full standard deviation change in grit resulted in 0.118 standard deviation change in well-being. The findings of the *t*-test (*t* = 3.435, *p* < 0.05) showed that the beta value of 0.188 enjoyed statistical significance. For the second model, the beta value of 0.337 specified that one full standard deviation variation in Grit resulted in 0.337 standard deviation variation in classroom enjoyment. The findings of the *t*-test (t = 6.414, *p* < 0.05) showed that the beta value of 0.188 enjoyed arithmetical significance.

## Discussion

The present study investigated the impacts of grit on learners' well-being and enjoyment in language education in the Chinese EFL context. Results indicate that grit was a momentous predictor of learners' well-being and enjoyment. It proved that grit can reinforce well-being and classroom enjoyment in the classroom. Hypothetically, these results support an emergent view of the role that PP can have in SLA (Wang et al., 2021). In this way, the advent of foreign language enjoyment (Dewaele and MacIntyre, 2014) set the stage for employing PP in language learning, and then other positive issues were identified in this line of research. For instance, enjoyment arouses attainment in action, which lowers the anxiety in learners and consequently brings about advances in life (Dewaele and Dewaele, 2017). Regarding grit, it can be stated that people with a high degree of grit are supposed to be capable of increasing their capabilities since they have greater focus and are less dejected by problems and obstacles (Credé et al., 2017). This proposes that grittier students, who sustain persistent effort in cultivating their language ability, are expected to be more engaged in the class. It could also be considered from the perspective that learners with a higher level of perseverance seem to work vigorously at challenging tasks, and through this enjoy their classes. This is somewhat reinforced by earlier studies (Duckworth et al., 2011) that have reported that gritty learners tend to work more and to perform better when facing difficulties. Based on the self-regulation theory of Bandura's theory (1991 as cited in Wei et al., 2019), students assess their own dispositions, thus eliciting positive or negative emotive retorts. When they try hard in the process of language learning, they tend to assess their own behaviors, thus generating positive emotive feedback, such as academic engagement (Derakhshan et al., 2019; Zheng, 2021). The findings of this research are in line with Vainio and Daukantaite's (2016) study that proved the predictive role of grit in predicting well-being. The meaningful relation between grit and well-being proved in this research are along the lines of research that discovered grit's correlation with ideas including pleasure and the individual's appraisal of life satisfaction leading to psychological well-being (Singh and Jha, 2008), and this is in line with the study carried out by Von Culin et al. (2014) which concluded that well-being is developed in gritty individuals through their trend to hunt for engagement as a main component of the well-being.

Furthermore, the results of the current paper support those of Wei et al. (2019) who concluded that there is a connection between grit and enjoyment, and also between grit and the language performances of learners. The results of this study are congruent with those (e.g., Li C. et al., 2018; Jin and Zhang, 2019) which proved how the significant role of PP variables such as enjoyment correlates with language learning development. The findings of this research support that carried out by Mierzwa (2019) who concluded that those learners who are interested in language education try to achieve higher goals in their life that are in line with the theory of grit. Another significant justification of the current study is that classroom enjoyment can be taken in light of Fredrickson's (2001) broad-and-build theory, that based on this theory when learners are in a positive classroom setting, they may have more enthusiasm in this process. As MacIntyre and Gregersen (2012) pointed out that constructive emotions may make students have more resilience, it seems reasonable that this positive atmosphere in the classroom may drive students to show more perseverance of effort, which is one of the main components of grit. The findings indicate that more enjoyment is related to grit, and that is in line with the study done by Dewaele et al. (2016) who declared that enjoyment is associated with low apprehension, though both can happen simultaneously. In addition, it is revealed that enjoyment and grit are mutually reinforcing-in which grit can be the predictor of enjoyment, and that it can be deemed that enjoyment encourages learners to keep on their path in language learning that consequently may decrease the anxiety of learners, which is in agreement with Dewaele and MacIntyre (2014).

## Conclusion and Implications

In line with the review of literature, many academics have proposed the influential role in language learning of the inner qualities of an individual such as personality traits like grit, which can be taught and developed. Investigating how grit conceives an individual's well-being and enjoyment in the process of language education is the focus of this study, which may bring further pedagogical developments. Indeed, realizing the function of grit may suggest a new viewpoint for language educators and students, and material developers.

Referring to these results regarding pedagogical suggestions for teachers, improving learners' sense of having a strong core of determination might allow them to deal with problems and challenges in language education. Learners were more likely to carry out challenging tasks, and less probable to give up after failure, and thus flourished and achieved greater results. People could not only increase their achievements over the course of their lives but also improve their well-being through constructing the use of engagement to become grittier. In addition, the teacher can incorporate grit into the language learning context as a practical technique to take full advantage of language use and finally improve learners' well-being. Educators can give speeches about the prominence of “effort” as a component of grit in the procedure of language education. Likewise, educators are recommended to be more mindful of learners' emotional states, since enjoyment may have fundamental roles in language education, students' feelings of enjoyment should be increased, which sequentially may decrease degrees of apprehension and result in better language learning.

Educators should be sociable, humorous, and caring, and attempt to establish innovative and stimulating classroom tasks that connect learners' language levels and well-being (Oxford, 2017; Dewaele et al., 2019). The abilities and optimism of the educator together with efficient and encouraging teaching tasks may support building and constructing a positive atmosphere in the classroom and increasing students' engagement, which, in sequence, will bring about better presentation (Yu et al., 2019; Wang et al., 2021). So, giving more attention, providing constructive support, as well as making motivating remarks may develop learners' feelings of approval, which could enhance their interactions with their friends in the language setting and eventually stimulate their sense of well-being and their enjoyment.

The outcomes of the present study could be put into practice in the development of classroom tasks. Students should be interested in the kind of tasks they find enjoyable, and the teacher could assist them in finding enjoyment in tasks that they might not relish. Enjoyment can increase distinct cognitive capitals and make learners' learning well-organized, and consequently, it can help them obtain constructive control, lower the burden on students, and stimulate their awareness in language education (Piniel and Albert, 2018).

Moreover, as it is eminent that learning a language is a process susceptible to apprehension, which is a danger to learners' levels of enjoyment, language teachers should build a less aggressive atmosphere in the language classroom. Nowadays with the advent of technologies, technology such as audio and videotape resources should be integrated into classroom syllabus to be implemented by teachers, to help construct a positive setting for learners in the class (Peng, 2019). To this end, material developers should try to design some tasks for language settings which improve grit, and these tasks may be prepared to inspire learners to arrange long-term objectives grounded on their well-being and enjoyment. In addition, to build a positive and peaceful language education situation, the institute or faculty should decrease the rate of tests (Jiang and Dewaele, 2019) and let teachers be free and autonomous in the language evaluation process. Indeed, planning sessions and beginning a discussion with faculty memberships about the significance of grit scopes can support learners' route toward accomplishment.

While the emphasis of the current research was put on discovering learners' grit and how it is correlated with the learners' well-being and their enjoyment, language educators' grit and how it influences their motivation and teaching experiences should not be ignored; more study can be done in this domain to focus on teacher's grit as well. The obtained findings contribute to establishing the indirect effect of the psychological bases of PP as a whole, and its constituents such as enjoyment and well-being; more studies can be done to focus on other variables such as confidence, efficacy, resilience, and positivity which seem to be related to grit and academic performance. Further studies will examine facilitating or controlling visible behaviors of grittier learners that will come up with academic growths of grit and to the progress of grit-improving intervention platforms. Implementation of longitudinal-type research is recommended since on the one hand, time is one of the important factors to obtain more obvious and beneficial data from participants (Elahi Shirvan and Taherian, 2018; Saito et al., 2018), and on the other hand, the grit scale is aimed to measure a person's desire and perseverance for long-standing objectives. It is not clear if improving grit is possible through treatment instructions or not, although it is apparent that societal and individual abilities are susceptible to treatment instructions, as it is proposed by some researchers (Durlak et al., 2010; Paunesku et al., 2015) that grit mediations may have some constructive consequences. That is to say, it may be possible to cultivate methods or tactics designed to aid persons, principally language learners, to increase grit and, feasibly, their well-being and enjoyment too. Also, the contestants of the present study were recruited from 28 universities in China, so the results may not be generalizable to other populaces. Forthcoming studies should observe the interactions between issues studied in this research across various types of students, cultures, or age groups.

## Data Availability Statement

The original contributions presented in the study are included in the article/supplementary material, further inquiries can be directed to the corresponding author.

## Ethics Statement

The studies involving human participants were reviewed and approved by Pingdingshan University Research Ethics Committee. The patients/participants provided their written informed consent to participate in this study.

## Author Contributions

PY conceptualized, collected data, analyzed data, and drafted the manuscript to submit it to Frontiers in Psychology.

## Conflict of Interest

The author declares that the research was conducted in the absence of any commercial or financial relationships that could be construed as a potential conflict of interest.

## Publisher's Note

All claims expressed in this article are solely those of the authors and do not necessarily represent those of their affiliated organizations, or those of the publisher, the editors and the reviewers. Any product that may be evaluated in this article, or claim that may be made by its manufacturer, is not guaranteed or endorsed by the publisher.
